# Association of cognitive function with frailty, nutritional status, and quality of life in older adults with mild cognitive impairment

**DOI:** 10.1371/journal.pone.0332377

**Published:** 2025-09-25

**Authors:** María del Mar Carcelén-Fraile, Anabel Melguizo-Garín, Aday Infante-Guedes, Raquel Medina-Ramírez, Sandra Denche-Gil, Agustín Aibar-Almazán, Yolanda Castellote-Caballero, María del Carmen Carcelén-Fraile

**Affiliations:** 1 Department of Health Sciences, Faculty of Health Sciences, University of Jaén, Jaén, Spain; 2 Department of Social Psychology, Social Work and Social Services and Social Anthropology. University of Málaga,; 3 Department of Health Sciences, Faculty of Health Sciences, University of Atlántico Medio, Las Palmas de Gran Canaria, Spain; 4 University of Las Palmas de Gran Canaria, Spain. Soc-Dig research group. Las Palmas de Gran Canaria, Spain; 5 Department of Education Sciences, Faculty of Social Sciences, University of Atlántico Medio, Las Palmas de Gran Canaria, Spain; University of Thessaly Faculty of Medicine: Panepistemio Thessalias Tmema Iatrikes, GREECE

## Abstract

**Background:**

In older adulthood, mild cognitive impairment is an intermediate stage between normal aging and dementia, making its detection crucial. Therefore, this study aims to analyze the associations between cognitive function and frailty, nutritional status, and quality of life in older adults with mild cognitive impairment.

**Methods:**

This work was conducted through a cross-sectional, analytical study involving 129 adults diagnosed with mild cognitive impairment, with a mean age of 68.07 ± 4.22. For cognitive assessment, the Mini-Mental State Examination (MMSE), Montreal Cognitive Assessment (MoCA), Isaac Verbal Fluency Test, Trail Making Test (TMT), D2 Test of Attention (D2 Test), and Digit Symbol Substitution Test (DSST) were completed; clinical and functional status was assessed using the frailty (FRAIL), Mini Nutritional Assessment (MNA), and life quality 36-Item Short Form Survey (SF-36) questionnaires.

**Results:**

Regarding overall cognitive performance, the presence of mild cognitive impairment was confirmed in the sample, as was the slowing of executive functions. Regarding selective attention, participants obtained an average of 138.30 points [SD = 4.30] in the D2 test, while the average score for processing speed measured using the DSST was 43.60 [SD = 3.50]. Regarding clinical and functional variables, the average FRAIL score was 2.26 [SD = 1.67], suggesting a high prevalence of frailty and pre-frailty; the average nutritional status was 27.91 [SD = 1.88], a range of adequate nutritional status. Finally, quality of life showed an average of 61.40 [SD = 14.87], indicating a moderate level.

**Discussion:**

This study shows that frailty, nutritional status, and quality of life are closely related to mild cognitive impairment. These results reinforce the need for early and multidimensional interventions that contribute to preserving the quality of life.

## Introduction

Population aging is a global phenomenon that has gained increasing relevance in recent decades [1]. Increasing life expectancy and declining birth rates have contributed to a sustained increase in the number of older adults [2]. This demographic shift brings with it significant public health challenges, including an increase in the prevalence of neurodegenerative diseases and cognitive disorders [3]. In particular, mild cognitive impairment [MCI] has become a topic of growing interest due to its high frequency in the elderly population and its potential progression to more severe forms of dementia, such as Alzheimer’s disease [4]. Mild cognitive impairment is defined as an intermediate stage between normal cognitive aging and dementia, characterized by a decline in one or more cognitive domains, such as memory, language, or attention, that does not significantly interfere with daily activities [5]. People with MCI retain relatively intact functional autonomy, although they may experience difficulties with more complex tasks [6]. This condition is heterogeneous in its clinical presentation, and early identification is crucial for implementing interventions that can delay or prevent its progression [7].

Several neuropsychological factors and variables have been associated with mild cognitive impairment underlying neurodegenerative processes and is useful for both early diagnosis and monitoring the progression of MCI [8]. Accurate quantification of this impairment allows MCI to be differentiated from normal aging changes and to establish possible trajectories toward dementia [9]. For example, decreases in the ability of verbal fluency are often among the first cognitive manifestations in people with MCI [10]. Other variables such as physical frailty have also been shown to be associated with mild cognitive impairment. Frailty manifests as a decrease in physiological reserve and stress resistance, muscle weakness, unintentional weight loss, fatigue, and decreased walking speed [11]. Its presence affects mobility and functional independence and interact with neurological processes and increase vulnerability to cognitive deficits [12].

Nutritional status is another key factor, as nutritional deficiencies in micronutrients and total energy can negatively impact brain structure and function. Malnutrition or undernutrition in older adults is associated with brain atrophy, reduced gray matter volume, and altered neurotransmission. Furthermore, specific deficiencies such as those of vitamin B12, folic acid, and essential fatty acids have been directly linked to the risk of developing MCI. Finally, quality of life, understood as the subjective perception of physical, psychological, and social well-being, is often compromised in people with MCI [13]. This reduction in quality of life is not only due to the cognitive limitations themselves, but also assessing this component allows us to better understand the functional and emotional impact of MCI on the lives of older adults [14].

Therefore, the present study aims to analyze the association between cognitive function, assessed through indicators such as verbal fluency, executive functions, attention, concentration, and processing speed, and related factors such as frailty, nutritional status, and quality of life in older adults with mild cognitive impairment.

## Materials and methods

### Design and Participants

This cross-sectional, analytical study included 129 older adults diagnosed with MCI, with a mean age of 68.07 ± 4.22 years (62% women and 38% men). The recruitment period for this study started on January 14th, 2025, and ended on February 13th, 2025. The study received approval from the Atlántico Medio University Ethics Committee (CEI05–012) and was conducted in compliance with the ethical principles established in the Declaration of Helsinki. All participants provided written informed consent before being included in the study.

**Fig 1:**
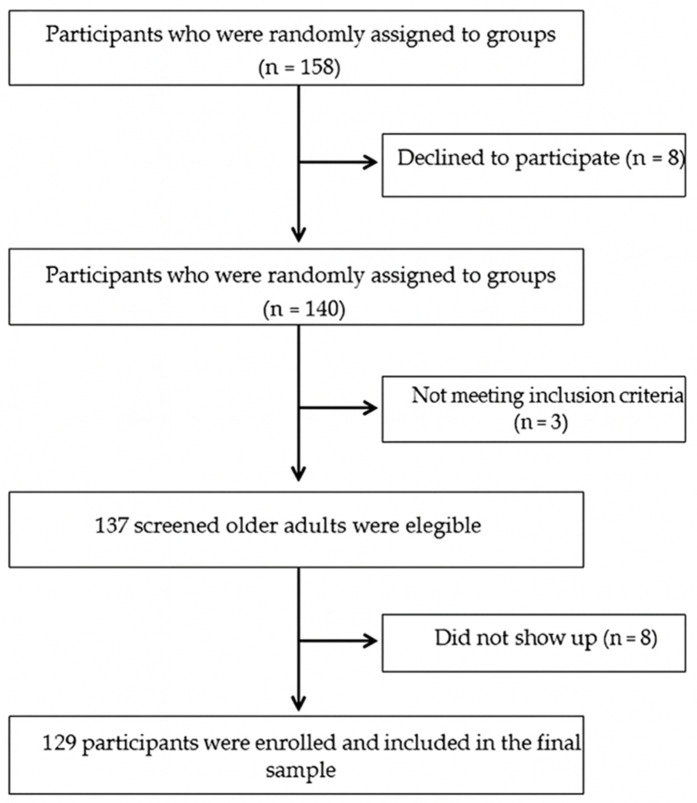
Flowchart of the study design.

The inclusion criteria required that subjects: (i) be 60 years of age or older, regardless of gender; (ii) score less than 24 points on the MMSE, which is indicative of MCI; (iii) had the necessary skills to understand and follow instructions, respond appropriately to assessment instruments, and participate in physical testing; and (iv) had signed the informed consent form. Individuals were excluded from the study if: (i) they had vestibular pathologies or other disorders affecting balance; (ii) they were under pharmacological treatment with medications that could alter the functioning of the central nervous system, coordination, or balance, such as anxiolytics, antidepressants, vestibular sedatives, or drugs aimed at improving cognition or vestibular function; and [iii] they suffered from severe visual disturbances that could not be corrected with optical devices or surgical interventions. These conditions included advanced macular degeneration, severe diabetic retinopathy, advanced glaucoma, and other visual diseases that seriously compromised peripheral or central vision (Fig 1).

### Instruments

Demographic information, including age, educational level, marital status, and occupation, was collected by specifically trained interviewers. Body mass index was obtained by dividing weight in kilograms by height in meters squared. A high-precision digital scale manufactured (Tefal®9, and a T201 T4 adult height rod (Asimed®) were used. Waist circumference was measured with a 1.5-meter flexible tape measure (Lufkin®). The measurement was taken at the midpoint between the last rib and the iliac crest, with participants in an upright position. To assess cognitive function and other clinical variables, a battery of psychometric instruments and standardized tests validated for use in older adults were administered based on cognitive, clinical and functional status:

*Mini-Mental State Examination (MMSE)* [15]: It assesses cognitive performance through functions such as temporal and spatial orientation, attention, calculation, immediate and delayed memory, language, and visuospatial construction skills. The total score ranges from 0 to 30, with scores equal to or greater than 27 indicating normal functioning; between 24 and 26, suspected impairment; between 12 and 24, moderate impairment; and below 12, severe impairment or dementia.

*Montreal Cognitive Assessment (MoCA) [*16]: This was used to detect mild cognitive impairment with domains such as memory, attention, executive functions, language, visuospatial skills, abstraction, and orientation. The maximum score is 30, and scores below 26 suggest cognitive impairment.

*Isaac Verbal Fluency Test* [17]*:* It measures semantic verbal fluency by asking the participant to name as many items as possible within a category [animals, fruits, colors, and cities] in 60 seconds. Each category is worth a maximum of 10 points, with a maximum total score of 40. It is considered a useful test for exploring semantic activation and verbal speed.

*Trail Making Test (TMT)* [18]: This test includes two parts. Part A (TMT-A) assesses visual attention, processing speed, and sequential tracking by connecting numbers in ascending order. Part B (TMT-B) measures executive functions by alternating between numbers and letters in sequential order.

*Digit Symbol Substitution Test (DSST)* [19]*:* assesses psychomotor processing speed. In this task, participants must match numbers with corresponding symbols within a limited time, copying as many pairs as possible. The total score corresponds to the number of symbols correctly matched in 90 seconds, with 60 being the maximum possible. It is sensitive to cognitive changes associated with aging and cognitive decline.

*FRAIL Questionnaire* [20]*:* This test assesses frailty level using five items exploring fatigue, endurance, walking ability, chronic diseases, and unintentional weight loss. Each item is answered “yes” or “no” (one point if yes). Scores range from 0 to 5, with 0 indicating no frailty, 1–2 indicating pre-frailty, and ≥3 indicating frailty.

*Mini Nutritional Assessment (MNA)* [21]: This tool evaluate nutritional status using 18 items grouped into four sections: anthropometric measurements, global assessment [comorbidity, mobility, medications], dietary assessment [meal patterns and fluid intake], and self-perceived health and nutrition. The maximum score is 30, where ≥24 indicates adequate nutritional status, between 17 and 23.5 suggests risk of malnutrition, and <17 indicates established malnutrition.

*Short Form-36 Health Survey* [22]*:* The validated Spanish version of the SF-36 was used to measure perceived quality of life. It includes 36 items distributed across eight dimensions: physical function, role physical, bodily pain, general health, vitality, social function, role emotional, and mental health. It also provides two summary components: physical component (PCS) and mental component (MCS). Scores are transformed to a scale of 0–100, with higher values reflecting better quality of life.

### Sample size calculation

The sample size and statistical power calculations were performed using G*Power software, version 3.1.9.2 [23]. A 95% confidence level and a 6% margin of error were established as reference parameters. Based on these criteria, it was determined that a minimum of 125 participants was sufficient to achieve 80% statistical power. Since the sample obtained met these requirements, no corrections for potential dropouts or losses were necessary. Participant selection was performed using a simple randomization procedure. Everyone within a previously consolidated list was assigned an identification number, and a random process was subsequently applied to invite 125 people to participate in the study. This method ensured equitable distribution and reduced the risk of selection bias.

### Data analysis

The collected data were analyzed using IBM SPSS Statistics, version 24.0. First, descriptive analyses were performed for all study variables, including means, standard deviations, ranges, and frequencies, to characterize the sample and verify data distribution. Subsequently, Pearson bivariate correlation analyses were conducted to explore associations between cognitive performance (MMSE, MoCA, verbal fluency tests, executive functions, attention, and processing speed) and clinical and functional variables: frailty (FRAIL), nutritional status (MNA), and quality of life (SF-36). Correlations were interpreted based on both magnitude and statistical significance, with a significance threshold of p < 0.05. Finally, multiple linear regression analyses using the forced entry method (enter) were performed to examine the predictive value of cognitive and sociodemographic variables (age and sex) on the dependent variables of interest: frailty, nutritional status, and quality of life. To analyse which factors are associated with the clinical and functional status of the participants, multiple linear regression models were performed. The dependent variables were quality of life (SF-36), nutritional status (MNA) and frailty (FRAIL scale). Independent variables included different measures of cognitive performance (MMSE, MoCA, verbal fluency, attention, processing speed, and executive functions), as well as age and sex. These models allowed us to explore which cognitive and sociodemographic variables best predict the well-being of older adults with mild cognitive impairment. The assumptions of linearity, independence of residuals, homoscedasticity, and normality were checked in each model by inspecting standardized residual plots and statistical tests. Results are expressed as unstandardized regression coefficients (B), standardized beta coefficients (β), t-values, and significance levels (p). The coefficient of determination (R²) and adjusted R² are also reported, as indicators of the percentage of variance explained by each model.

## Results

The results are presented in a sequence that allows for a progressive understanding of the characteristics of the sample and the relationships between the variables studied. First, descriptive analyses are presented to determine the mean values and distribution of the main sociodemographic, cognitive, clinical, and functional variables. Next, the correlation analyses are shown, which allow us to identify the associations between cognitive performance and variables such as frailty, nutritional status and quality of life. Finally, the multiple regression models are presented, which allow us to analyse which cognitive and sociodemographic variables best predict functional status and perceived well-being in this population.

### Descriptive statistics

Descriptive analyses allowed us to characterize the sample in relation to the cognitive, clinical, and functional variables studied. The mean age of the participants was ≈ 68years old (SD = 4.22), with a range between 60 and 78 years old. Regarding gender, 62% of the sample were women and 38% men. No statistically significant differences were found by gender in any of the cognitive, functional, or quality of life variables evaluated, including MMSE, MOCA, frailty, and perceived quality of life (all p > 0.05).

Regarding global cognitive performance, the mean score on the MMSE was 20.97 (SD = 1.31) and the MoCA was 21.45 (SD = 1.68). The mean score on the Isaacs Verbal Fluency Test was 17.19 (SD = 1.73).

For executive functions, the mean time to complete the TMT-A was 80.53 seconds (SD = 9.64), and for the TMT-B it was 169.72 seconds (SD = 65.93). Regarding selective attention, participants obtained an average score of 138.30 points (SD = 4.30) on the D2 test, while the mean score for processing speed measured using the DSST was 43.60 (SD = 3.50).

In terms of clinical and functional variables, the mean score on the FRAIL frailty scale was 2.26 (SD = 1.67). Regarding nutritional status, assessed using the MNA, the mean score was 27.91 (SD = 1.88), mostly within the range of adequate nutritional status. Finally, perceived quality of life, assessed using the SF-36, showed a mean of 61.40 (SD = 14.87). (Table 1)

**Table 1:** Descriptive characteristics of the sample and mean scores on cognitive, clinical, and functional variables (n = 129).

Outcome	Mean	SD	Min	Max
Age [years]	68.07	4.22	60	78
MMSE	20.97	1.31	19	23
MoCA	21.45	1.68	19	25
Verbal Fluency [Isaac]	17.19	1.73	14	20
TMT-A [seconds]	80.53	9.64	64	99
TMT-B [seconds]	169.72	65.93	38	300
Attention [D2]	138.3	4.3	128	145
DSST	43.6	3.5	35	50
Frailty [FRAIL]	2.26	1.67	0	5
Nutritional Status [MNA]	27.91	1.88	24	30
Quality of life [total SF-36]	61.4	14.87	45	90

Table Notes: MMSE = Mini-Mental State Examination; MoCA = Montreal Cognitive Assessment; TMTA/B = Trail Making Test A and B; D2 = Selective Attention Test; DSST = Digit Symbol Substitution Test; MNA = Mini Nutritional Assessment; SF-36 = Short Form-36 Health Survey. FRAIL = Frailty Scale. Higher scores on the TMTA and TMTB indicate poorer performance. SD = Standard deviation. Results are expressed as unstandardized regression coefficients (B), standardized beta coefficients (β), t-values, and significance levels (p).

### Correlations between variables

Moderate positive correlations were observed between the MMSE and nutritional status (r = .28, p < 0.01), as well as between the MoCA and quality of life (r = 0.21, p < 0.05). Significant correlations were also found between verbal fluency (Isaac) and quality of life (r = 0.26, p < 0.01). Regarding specific executive and cognitive functions, significant correlations were found between processing speed (DSST) and nutritional status (r = 0.28, p < 0.01), as well as with quality of life (r = 0.25, p < 0.01. Similarly, attention (D2) correlated positively with MNA (r = 0.26, p < 0.01) and quality of life (r = 0.24, p < 0.01). Although the correlations between cognitive performance and frailty were weaker, significant associations were identified with the MMSE (r = 0.19, p < 0.05), MoCA (r = .21, p < 0.05), and TMT-B (r = .18, p < 0.05). (Table 2)

**Table 2:** Pearson correlations between cognitive variables, nutritional status, frailty, and quality of life.

Outcomes	MMSE	MoCA	Isaac	TMTA(sec)	TMTB (sec)	D2	DSST	Frailty	MNA	Quality of life
**MMSE**	—	0.95^**^	0.94^**^	0.96^**^	0.97^**^	0.96^**^	0.96^**^	0.19^*^	0.28^**^	0.22^*^
**MoCA**		—	0.97^**^	0.96^**^	0.97^**^	0.97^**^	0.97^**^	0.21^*^	0.23^**^	0.21^*^
**Isaac**			—	0.91^**^	0.95^**^	0.97^**^	0.96^**^	0.23^**^	0.19^*^	0.26^**^
**TMTA**				—	0.99^**^	0.96^**^	0.97^**^	0.15	0.24^**^	0.20^*^
**TMTB**					—	0.98^**^	0.99^**^	0.18^*^	0.24^**^	0.23^**^
**D2**						—	0.99^**^	0.21^*^	0.26^**^	0.24^**^
**DSST**							—	0.20^*^	0.28^**^	0.25^**^
**Frailty**								—	0.07	0.09
**MNA**									—	0.06
**Quality of life**										—

Table Notes: 95% CI. MMSE = Mini-Mental State Examination; MoCA = Montreal Cognitive Assessment; TMTA/B = Trail Making Test A and B; D2 = Selective Attention Test; DSST = Digit Symbol Substitution Test; MNA = Mini Nutritional Assessment; SF-36 = Total Quality of Life.

** p < 0.05.

* p < 0.01. Higher scores on TMT-A and TMT-B indicate lower executive performance.

Table 2 shows the bivariate correlations between the different measures of cognitive functioning and the clinical and functional variables considered in the study: frailty, nutritional status, and quality of life. This analysis provides a general overview of how the variables evaluated relate to each other, identifying positive or negative associations and guiding subsequent analyses on prediction and association between the different factors.

All assumptions for Pearson correlations and multiple regression (linearity, independence of errors, homoscedasticity, normality, and absence of multicollinearity) were checked and met. Variance inflation factor (VIF) values ranged between X and Y, indicating no problematic collinearity.

### Multivariable analysis

The multiple regression model predicting quality of life (SF-36) from cognitive variables, age, and sex was significant, F(9, 119) = 2.45, p = 0.013, explaining 8.2% of the variance (adjusted R² = 0.089.

Within the model, the variables that approached significance were the MoCA score (standardised β = −0.835, p = 0.069), verbal fluency (standardised β = 0.870, p = 0.060), and processing speed (DSST; standardised β = 1.170, p = 0.083). (Table 3).

**Table 3:** Multiple linear regression model for predicting quality of life (SF-36).

Outcomes	B	β	t	p
Age	−0.144	−0.037	−0.42	0.674
Sex [1 = women]	3.876	0.127	1.46	0.147
MMSE	−2.504	−0.220	−0.59	0.556
MoCA	−7.380	−0.835	−1.84	0.069
Verbal Fluency	7.463	0.870	1.90	0.060
TMTA	−0.514	−0.333	−0.45	0.652
TMTB	0.061	0.272	0.28	0.784
Attention [D2]	−2.348	−0.679	−1.07	0.287
DSST	4.967	1.170	1.75	0.083

Tables notes: Model: 95% CI. F[9, 119] = 2.45, p = 0.013, adjusted R² = 0.08 Note: Dependent variable: total score on the SF-36. MMSE = Mini-Mental State Examination; MoCA = Montreal Cognitive Assessment; DSST = Digit Symbol Substitution Test; TMTA/B = Trail Making Test A and B; D2 = Selective Attention Test. Sex coded as 1 = female, 2 = male. p < 0.05. *p < 0.01. Results are expressed as unstandardized regression coefficients (B), standardized beta coefficients (β), t-values, and significance levels (p).

The model for predicting nutritional status (MNA) was statistically significant, F(9, 119) = 4.02, p < 0.001, explaining 17.5% of the variance (adjusted R² = 0.18).

Within the model, significant predictors were the MMSE (standardised β = 0.927, p = 0.010), the DSST (β = 1.587, p = 0.014), and, inversely, verbal fluency (standardised β = −1.549, p = 0.001). (Table 4).

**Table 4:** Multiple linear regression model for predicting nutritional status (MNA).

Outcomes	B	β	t	p
Age	0.032	0.066	0.79	0.434
Sex [1 = women]	−0.229	−0.060	−0.72	0.471
MMSE	1.332	0.927	2.63	0.010
MoCA	0.479	0.429	1.00	0.321
Verbal Fluency	−1.677	−1.549	−3.57	0.001
TMTA	−0.228	−1.173	−1.68	0.096
TMTB	−0.012	−0.430	−0.46	0.647
Attention [D2]	0.186	0.427	0.71	0.479
DSST	0.850	1.587	2.50	0.014

Tables notes: Model: 95% CI. F[9, 119] = 4.02, p < 0.001, adjusted R² = 0.18 Note: Dependent variable: Mini Nutritional Assessment total score. MMSE = Mini-Mental State Examination; MoCA = Montreal Cognitive Assessment; DSST = Digit Symbol Substitution Test; TMTA/B = Trail Making Test A and B; D2 = Selective Attention Test. Sex coded as 1 = female, 2 = male. p < 0.05. *p < 0.01. Results are expressed as unstandardized regression coefficients (B), standardized beta coefficients (β), t-values, and significance levels (p).

The model for frailty was marginally significant, F(9, 119) = 1.77, p = 0.082, with an adjusted R² of 5.1%. Although it did not reach statistical significance at the conventional level, the results show some associations that merit attention.

The age variable was the one that came closest to the significance threshold (standardised β = 0.168, p = 0.064), indicating a tendency for older ages to be associated with higher levels of frailty. None of the cognitive variables were found to be significant individual predictors (Table 5).

**Table 5:** Multiple linear regression model for the prediction of frailty (FRAIL scale).

Outcomes	B	β	t	p
Age	0.073	0.168	1.87	0.064
Sex [1 = women]	0.237	0.069	0.78	0.435
MMSE	0.148	0.115	0.31	0.761
MoCA	0.476	0.480	1.04	0.301
Verbal Fluency	−0.096	−0.100	−0.22	0.831
TMTA	−0.118	−0.681	−0.91	0.365
TMTB	−0.013	−0.496	−0.49	0.623
Attention [D2]	0.313	0.806	1.25	0.214
DSST	0.039	0.083	0.12	0.904

Table notes: Model:: 95% CI. F[9, 119] = 1.77, p = 0.082, adjusted R² = 0.05 Note: Dependent variable: total score on the FRAIL scale. MMSE = Mini-Mental State Examination; MoCA = Montreal Cognitive Assessment; DSST = Digit Symbol Substitution Test; TMTA/B = Trail Making Test A and B; D2 = Selective Attention Test. Sex coded as 1 = female, 2 = male. p < 0.05. *p < 0.01. Results are expressed as unstandardized regression coefficients (B), standardized beta coefficients (β), t-values, and significance levels (p).

## Discussion

The objective of this study was to examine the relationship between cognitive function, frailty, nutritional status, and quality of life in older adults with mild cognitive impairment. The findings show that these four domains are closely interrelated, suggesting that cognitive decline does not occur in isolation but is accompanied by a series of factors that simultaneously impact on the overall health and well-being of this population.

One of the most notable findings of this study is that global cognitive performance, assessed by using widely used tests such as the MMSE and the MoCA, is positively associated with higher perceived quality of life. This result suggests that a higher level of overall cognitive functioning has clinical implications in terms of autonomy and functionality, which directly influences the subjective perception of well-being in older adults with MCI [24].

Specifically, executive functions such as processing speed and selective attention also showed favorable associations with improved perceived quality of life. These findings reinforce the idea that, beyond global cognitive functioning, certain executive skills play a significant role in the experience of well-being. This includes moderate verbal ability within the possible range (maximum = 40), possibly due to their role in solving everyday problems, decision-making, and emotional self-regulation [25]. These results are in line with scientific literature, which has consistently emphasized the importance of cognitive status as one of the factors contributing to subjective well-being and functionality in daily life [26,27].

However, it is important to consider that the evidence [28] has not found a clear relationship between cognitive status and quality of life in older adults, suggesting that other factors, such as mental health, psychosocial environment, or illness awareness, may have a greater impact. Along these lines, Bonfiglio et al. [29] evaluated various cognitive functions that found that depressive symptoms had a greater determining weight in reported quality of life.

Despite these discrepancies, interesting clues were also observed in the present study, such as the fact that skills such as verbal fluency and processing speed could be positive predictors of quality of life. The regression model indicates a clear trend between cognitive processes and health perception. This suggests that greater expressive ability is related to a more positive perception of life status. This is particularly relevant because these functions are associated with communication skills, the ability to adapt quickly to new situations, and the ability to efficiently manage multiple stimuli-competences that can facilitate a subjective experience of greater control and satisfaction. Higher levels of verbal fluency and processing speed may be related to better quality of life, while a lower MoCA score, possibly associated with greater subjective burden, is linked to a worse perception of general health. In this sense, studies with larger samples and statistical power could provide more robust confirmation of these preliminary associations. Özge Saraçlı and colleagues [30], concluded that performance on the MMSE is a significant predictor of quality of life in older adults, reinforcing the idea that cognitive preservation should be a priority in intervention programs for people with MCI. Taken together, these results suggest that cognitive function, both globally and in specific domains, represents a key dimension to consider in a comprehensive approach to well-being in old age.

Nutrition has been established as a key modifiable factor in preventing and mitigating cognitive decline in older adults [31,32]. This evidence has generated growing interest in promoting nutritional education-based interventions that promote both brain health and general well-being in old age [33]. Several studies have shown that mediterranean diet pattern is not only associated with better results in cognitive function tests but also with a lower risk of developing neurodegenerative diseases such as Alzheimer’s [34]. Along these lines, the results obtained in the present study reinforce the evidence on the relationship between cognitive function and nutritional status. A significant and positive association was found between MMSE scores and processing speed, measured through the DSST, with nutritional status assessed through the Mini Nutritional Assessment (MNA). These results indicate that better global cognitive performance is associated with better perceptions of health and nutrition. Likewise, selective attention, measured with the D2 test, showed positive correlations with the MNA, suggesting that a more favorable nutritional status is consistently linked to better cognitive performance, not only globally, but also in specific executive domains.

Furthermore, cognitive performance on the MMSE and DSST showed significant predictive power for nutritional status, suggesting a potential bidirectional relationship between the two constructs. Interestingly, while higher performance on the MMSE and DSST is associated with better nutritional status, higher verbal fluency is paradoxically associated with lower MNA scores. On the one hand, good nutrition could facilitate the maintenance of cognitive functions by providing essential nutrients for neuronal function, synaptic plasticity, and protection against oxidative stress. On the other hand, greater cognitive integrity could facilitate healthier eating, thanks to greater functional autonomy, planning, and decision-making capacity. These findings align with previous research, such as that of Olivia Bornæs [35], which documented associations between mild cognitive impairment (MCI) and poorer nutritional status.

Regarding specific nutrients that may play a neuroprotective role, a 2018 systematic review by Andrea M. McGrattan et al. [36] identified several compounds with therapeutic potential for cognitive function, including B vitamins (particularly B6, B9, and B12), long-chain omega-3 fatty acids (DHA and EPA), and cocoa flavonols. These compounds have shown positive effects on functions such as memory, processing speed, and attention, by acting on mechanisms such as reducing inflammation, improving cerebral blood flow, and maintaining neuronal structure. The results of this study reaffirm the importance of early identification of nutritional risk, combined with personalized dietary interventions, as an effective strategy for preventing or slowing cognitive decline [37]. This evidence supports the need for multidisciplinary approaches in geriatric care, integrating neuropsychological assessment with nutritional monitoring [38].

Although frailty showed weaker associations with cognitive function measures in the present study compared with other variables analyzed, statistically significant relationships were identified with the MMSE, the MoCA, and the TMT-B. These associations, although smaller in magnitude, are clinically relevant and suggest that impaired executive functions, particularly those linked to planning, cognitive flexibility, and processing speed, may increase the physical and functional vulnerability of older adults with MCI. This frailty syndrome is influenced by a wide range of physical, psychological, social, and biological factors [39]. In this way, the vision of frailty as a dynamic and multidimensional phenomenon is reinforced, in which cognitive impairment represents only one of the multiple components [11].

These findings are consistent with existing scientific literature, which recognizes frailty as a complex syndrome in which cognitive impairment acts as a significant risk factor [40]. It is important to highlight that this relationship is bidirectional: on the one hand, physical frailty can precipitate or accelerate cognitive decline, and on the other, the presence of MCI can favor the onset of frailty by limiting physical activity, hindering health self-management, and increasing social isolation [41]. It should be noted that the sample in this study presented on average, overall cognitive performance below normative values, which confirms their characterization as a population with MCI. Even in these early stages of decline, negative impacts are evident not only on cognitive functioning but also on quality of life, nutritional status, and levels of frailty.

From a clinical perspective, this study emphasizes the importance of incorporating systematic assessments of cognitive status into comprehensive geriatric assessments. In turn, a multidisciplinary approach is proposed that includes not only the neuropsychological component but also nutritional, functional, and physical assessments, especially in older adults showing early signs of cognitive decline. This holistic approach can optimize therapeutic resources and improve long-term outcomes in this vulnerable population. However, some limitations of this study must be acknowledged. Cross-sectional design prevents establishing causal relationships and limits the ability to assess the progression of cognitive deficits and their impact on functionality. Furthermore, certain contextual and personal variables were not considered, such as educational level, socioeconomic status, frequency of physical activity, or the presence of chronic diseases, all factors with a potential influence on levels of frailty and cognitive function. The inclusion of these variables in future studies could allow for a more comprehensive analysis of the phenomenon. Executive functions, particularly attention and TMT performance, could be linked to perceived frailty in more powerful studies. For future research, we recommend opting for longitudinal designs that allow observation of the temporal evolution of frailty and cognitive decline, as well as evaluating the effectiveness of combined interventions. For example, programs that integrate cognitive stimulation, personalized nutritional interventions, and adapted physical activities could offer a comprehensive strategy to slow the progression of decline and preserve functional independence. Validation of these intervention models in specific clinical settings would provide valuable evidence for their application in the healthcare setting.

## Conclusion

This study provides solid evidence of the close relationship between cognitive function, quality of life, nutritional status, and frailty in older adults with mild cognitive impairment. The results highlight the importance of cognition, especially executive functions and processing speed, as key components in promoting overall health in old age. Consequently, the need to develop intervention strategies that integrate cognitive, nutritional, and physical approaches is reaffirmed.

## Supporting information

S1 Table Supplementary table analysis. The analysis of the independent variables is available in supplementary material.
(PDF)
