# Supplementary material for: Association of cognitive function with frailty, nutritional status, and quality of life in older adults with mild cognitive impairment
Source: PLoS One. 2025 Sep 25;20(9):e0332377. doi: 10.1371/journal.pone.0332377 (PMC12463238; doi:10.1371/journal.pone.0332377)
Supplement: S1 Table — The analysis of the independent variables is available in supplementary material. (PDF) [file pone.0332377.s001.pdf]

SUPPLEMENTARY MATERIAL

**Independent Samples Test**										
		Levene's Test for Equality of Variances		t-test for Equality of Means						
		F	Sig.	t	df	Sig. (2-tailed)	Mean Difference	Std. Error Difference	95% Confidence Interval of the Difference: Lower	Upper
MMSE	Equal variances assumed	,000	,993	,344	127	,732	,082	,237	-,388	,552
	Equal variances not assumed			,344	102,101	,731	,082	,237	-,389	,552
MOCA	Equal variances assumed	,342	,560	,974	127	,332	,297	,305	-,307	,901
	Equal variances not assumed			,998	109,359	,321	,297	,298	-,293	,888
ISAAC	Equal variances assumed	,147	,702	,994	127	,322	,313	,314	-,309	,934
	Equal variances not assumed			1,005	105,234	,317	,313	,311	-,304	,929
TMTA	Equal variances assumed	,152	,698	,529	127	,598	,928	1,754	-2,543	4,400
	Equal variances not assumed			,532	103,337	,596	,928	1,745	-2,533	4,389
TMTB	Equal variances assumed	,052	,819	,596	127	,552	7,152	11,991	-16,576	30,880
	Equal variances not assumed			,599	103,232	,550	7,152	11,933	-16,513	30,817
D2	Equal variances assumed	,073	,787	,623	127	,534	,488	,782	-1,060	2,035
	Equal variances not assumed			,625	102,493	,533	,488	,780	-1,059	2,034
DSST	Equal variances assumed	,002	,968	,737	127	,463	,469	,636	-,790	1,728
	Equal variances not assumed			,735	100,792	,464	,469	,638	-,797	1,734
FRAGILIDAD	Equal variances assumed	2,604	,109	-,375	127	,708	-,114	,304	-,715	,487
	Equal variances not assumed			-,362	90,045	,718	-,114	,315	-,740	,512
MNA	Equal variances assumed	,497	,482	,465	127	,643	,159	,341	-,517	,834
	Equal variances not assumed			,461	98,856	,646	,159	,344	-,524	,842
calidadvida	Equal variances assumed	5,214	,024	-1,057	127	,292	-2,851	2,696	-8,186	2,484
	Equal variances not assumed			-1,028	92,585	,307	-2,851	2,773	-8,358	2,657
